# Multiplex Fluorescence Melting Curve Analysis for Mutation Detection with Dual-Labeled, Self-Quenched Probes

**DOI:** 10.1371/journal.pone.0019206

**Published:** 2011-04-28

**Authors:** Qiuying Huang, Zanzan Liu, Yiqun Liao, Xiaoyun Chen, Yi Zhang, Qingge Li

**Affiliations:** 1 Engineering Research Center of Molecular Diagnostics, Ministry of Education, Department of Biomedical Sciences, School of Life Sciences, and the Key Laboratory of the Ministry of Education for Cell Biology and Tumor Cell Engineering, Xiamen University, Xiamen, Fujian, China; 2 Institute for Biomedical Research, Xiamen University, Xiamen, Fujian, China; St. Petersburg Pasteur Institute, Russian Federation

## Abstract

Probe-based fluorescence melting curve analysis (FMCA) is a powerful tool for mutation detection based on melting temperature generated by thermal denaturation of the probe-target hybrid. Nevertheless, the color multiplexing, probe design, and cross-platform compatibility remain to be limited by using existing probe chemistries. We hereby explored two dual-labeled, self-quenched probes, TaqMan and shared-stem molecular beacons, in their ability to conduct FMCA. Both probes could be directly used for FMCA and readily integrated with closed-tube amplicon hybridization under asymmetric PCR conditions. Improved flexibility of FMCA by using these probes was illustrated in three representative applications of FMCA: mutation scanning, mutation identification and mutation genotyping, all of which achieved improved color-multiplexing with easy probe design and versatile probe combination and all were validated with a large number of real clinical samples. The universal cross-platform compatibility of these probes-based FMCA was also demonstrated by a 4-color mutation genotyping assay performed on five different real-time PCR instruments. The dual-labeled, self-quenched probes offered unprecedented combined advantage of enhanced multiplexing, improved flexibility in probe design, and expanded cross-platform compatibility, which would substantially improve FMCA in mutation detection of various applications.

## Introduction

High throughput sequencing approaches have facilitated genome-wide discovery of mutations characteristic of various disease statuses [1]. Translation of these mutations-disease biomarkers into clinical diagnostics yet requires simple, rapid, and cost-effective mutation detection methods that have high multiplexing ability and cross-platform compatibility [2]. Homogeneous methods, exampled by real-time PCR, have proved to be very useful in mutation detection due to easy automation, high throughput, and low risk of post-PCR contamination [3], [4], [5]. Real-time PCR, however, encounters technical difficulties when multiple mutations from one sample need to be detected simultaneously in a single tube. Since each mutation needs a specific probe with a unique color, the number of distinguishable fluorophores and fluorescence detection channels in a fluorometric thermocycler becomes the bottleneck for multiplex detection. These limitations can be addressed by a post-PCR, probe-based fluorescence melting curve analysis (FMCA) procedure that allows detection of multiple mutations by a single probe based on melting temperature (T_m_) shifts [6]. The number of mutations detectable can be further increased if multiple probes each labeled with a different fluorophore are used (color multiplexing) [7]. In addition, unknown mutations can be scanned with FMCA by using a series of single-labeled probes complementary to the wild-type sequence [8]. Recently, even molecular haplotyping was achieved across a distance of 100 bp by FMCA using a looping-out design [9]. Consequently, FMCA has become a versatile tool for mutation detection [10], [11].

The increasing use of FMCA has also witnessed continuous evolution of its probe chemistry towards enhanced multiplexing, expanded flexibility, and reduced complexity. Since the first report of using Cy5-labeled primer and fluorescein-labeled probe combination, fluorescence resonance energy transfer (FRET) has become the dominant chemistry for FMCA. The primer-probe combination method was soon replaced with the dual hybridization probe approach, which is more amenable to multiplex detection [12]. To reduce the complexity of probe design, fluorescein-labeled probe [13] and unlabeled probes were developed for FMCA [14]. Alternative probes like HyBeacon [15], Biprobe [16], induced FRET (iFRET) [17], light emission modifiers [18], and dual-labeled probe of low T_m_ [19] were also reported. Recently, Pleiades probe has shown low background and high hybridization-triggered fluorescence when used for FMCA [20]. More recently, sloppy molecular beacon probes have been used to provide increased color multiplexing for FMCA [21].

Despite the aforementioned technical advancement, a combined merit of simplicity in probe design, cost-effectiveness in probe synthesis, high order color multiplexing, and cross-platform compatibility for FMCA remains to be achieved from one probe type. We investigated alternative real-time PCR probes in their potential for FMCA. We hypothesized that any probe that can exhibit fluorescence change upon thermal dissociation from their targets should be applicable to FMCA. We focused on those real-time PCR probes that are easy to design, cheap to synthesize, amenable to color multiplexing, and compatible to different platforms. Two self-quenched probes, TaqMan probe and shared-stem molecular beacons, met our criteria. After a thorough study on the experimental conditions for FMCA, we demonstrated that these two types of probes enable FMCA to be used for mutation scanning, mutation identification and mutation genotyping and confer cross-platform compatibility on major real-time PCR instruments.

## Results

### Dual-labeled, Self-quenched Probes for FMCA

**Figure 1:**
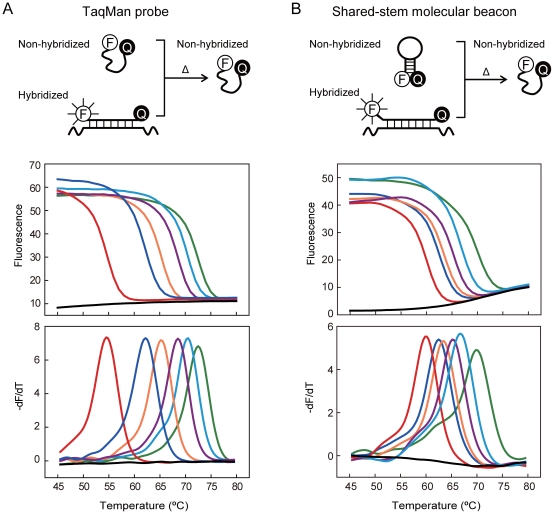
Denaturation of hybrids formed between probes and synthetic oligonucleotides. **A**) TaqMan probe. In aqueous solution, non-hybridized probe is weakly fluorescent at low temperature because the randomly coiled conformation causes fluorescence quenching due to the proximity of the fluorophore (F) and quencher (Q). The probe becomes strongly fluorescent when hybridized with its target. After thermal dissociation (Δ) from its target, the probe returns back to weakly fluorescent state. **B**) Shared-stem molecular beacons. Non-hybridized probe is basically non-fluorescent at low temperature due to the existence of hairpin structure that causes nearly complete quenching. Probe becomes strongly fluorescent when hybridized with its target. After thermal dissociation from its target, the non-hybridized shared-stem molecular beacon adopts a randomly coiled conformation similar to the TaqMan probe, and is weakly fluorescent. Melting curve plots (upper panel) of fluorescence (F) versus temperature (T) are transformed into melting peaks (lower panel) by plotting -dF/dT versus temperature. Different black lines represent targets with different mismatches to the hybridization probe, with fully matched wild-type target giving the highest T_m_ value (positioned at the far right side). The gray lines represent the results from NTC. The T_m_ value for each target is listed in Table 3 in the online Data Supplement.

TaqMan probe is a typical dual-labeled, self-quenched probe. A standard TaqMan probe is a linear oligonucleotide consisting of a fluorophore covalently attached to the 5′-end and a quencher at the 3′-end. The randomly coiled conformation enables fluorescence quenching unless the probe is either hybridized or digested [22]. Therefore, non-hybridized TaqMan probe is only weakly fluorescent but becomes strongly fluorescent when hybridized with its target. After denaturation from the probe-target hybrid, the probe returns back to its weakly fluorescent state (Figure 1A, top panel). We hybridized a typical TaqMan probe to 6 differently mismatched oligonucleotide targets and then examined the hybrids for thermal denaturation. The results showed that, as expected, fluorescence intensity of the hybrids decreased as temperature increased in a target-dependant manner (Figure 1A, middle panel), yielding different T_m_ value for each target derived from the melting peak (Figure 1A, bottom panel and Table S1). These results demonstrated the feasibility of TaqMan probe for FMCA.

Another type of dual-labeled, self-quenched probe, shared-stem molecular beacon, is a modified version of molecular beacon, which has its stem sequence partially or fully complementary to the target sequence [23]. Such modifications have showed higher hybridization efficiency but lower specificity [24]. Non-hybridized shared-stem molecular beacon is basically non-fluorescent at low temperature due to the existence of hairpin structure that causes nearly complete quenching. but becomes strongly fluorescent when hybridized with its target. After denaturation from the probe-target hybrid, non-hybridized shared-stem molecular beacon will have an open loop and, like TaqMan probe, adopt a randomly coiled configuration and becomes weakly fluorescent at high temperature (Figure 1B, top panel). Results obtained from shared-stem molecular beacon were similar to TaqMan probe (Figure 1B middle and bottom panels and Table S2). Of note, none of two probes displayed flat baseline fluorescence, which nevertheless exerted no influence on the melting peaks of the hybrids.

We further studied whether TaqMan probe with increased length would weaken the quenching efficiency and thus lead to poor FMCA signal. Four TaqMan probes of 26 nt, 30 nt, 36 nt, and 41 nt long were studied each with six differently matched targets (Table S3). The results showed that all probes could display melting curves with their targets (Figure S1). These results indicated that efficient fluorescence quenching exists between the terminally labeled fluorophore and quencher over a wide range of probe lengths. Since TaqMan probes designed for real-time PCR are usually within the above length range, we conclude that regular TaqMan probes can be used directly for FMCA.

### Optimization of PCR Conditions for FMCA

**Figure 2:**
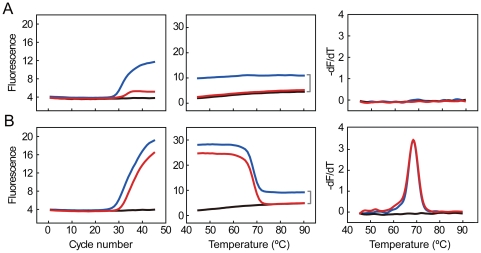
Comparison of symmetric and asymmetric PCR in FMCA. **A**) Symmetric PCR. **B**) Asymmetric PCR. A three-temperature cycling protocol was performed using either 5′-nuclease active Taq HS (blue lines) or 5′-nuclease-deficient Klentaq1 (red lines) DNA polymerase. Three data forms are given from left to right: amplification curves, meting curves, and negative derivative melting curves. Probe cleavage is shown by the fluorescence difference between the blue and black lines (indicated by a bracket). NTCs are shown in black lines.

It is widely recognized that TaqMan probe used in real-time PCR works in a hydrolysis way [25] and probe degradation was also observed in molecular beacons during real-time PCR [26]. Such features may reduce FMCA efficiency due to the lack of sufficient intact probes [11]. On the other hand, as an end-point detection format, FMCA would benefit from excess accumulation of single-stranded amplicons complementary to the probe and such amplicons can be readily generated by asymmetric PCR. We thus investigated the effects of Taq HS (with 5′-nuclease activity) versus Klentaq1 (without 5′-nuclease activity) and asymmetric versus symmetric PCR with both TaqMan and shared-stem molecular beacon probes. The results showed that symmetric PCR could generate typical amplification curves but it yielded low signals in the melting curves regardless of whether Taq HS or Klentaq1 was used (Figure 2A). In contrast, asymmetric PCR could generate melting curves as well as amplification curves in all cases even if probe cleavage occurred (Figure 2B). Similar results were obtained when two-temperature cycling protocol was used (Figure S2). Therefore, we conclude that asymmetric PCR enables TaqMan and shared-stem molecular beacon probes to be successfully used for FMCA. In the following examples, the two types of probes were not discriminated and used interchangeably.

### Multicolor FMCA for Mutation Scanning

**Figure 3:**
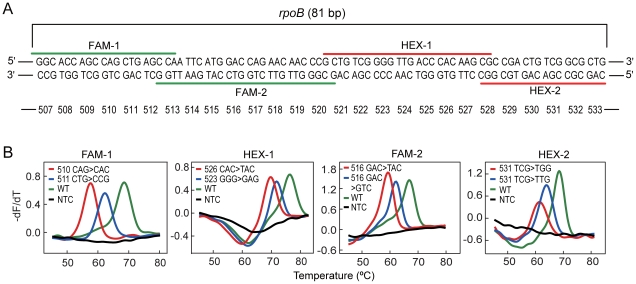
A 2-color FMCA assay for mutations scanning. **A**) The alignment of the four probes along the 81-bp region of *rpoB* gene of *M. tuberculosis*. Each probe is shown by its respective labeling fluorophore and the corresponding tube (e.g., FAM-1 stands for FAM-labeled probe in tube 1). The corresponding tri-nucleotide codons covered by the probes are shown under the probes. **B**) Representative results in the mutation scanning from clinical isolates. The derivative melting curves obtained from different detection channels are displayed according to the labeling fluorophores. In each channel, both wild-type (WT) curve and mutant curves are given. The mutant type is identified with mutation locations and mutation types, e.g., in FAM-1, 510>CAC indicates that codon 510 is mutated from CAG to CAC.

In the first proof-of-principle study, we designed an FMCA assay that allowed scanning for mutations randomly occurring within an 81-bp region of *rpoB* gene of *M. tuberculosis* that confer resistance to rifampin. Four probes in two reactions were positioned in a tiling format to cover the entire 81-bp region (Figure 3A). The assay was used to analyze 311 blind cultured sputum-positive tuberculosis samples, among which 137 were detected to harbor 23 different types of mutations, as confirmed by DNA sequencing of PCR amplicons (Table S4). The concordance rate between FMCA and sequencing results was 100%. Typical FMCA results of the frequent mutations were shown in Figure 3B. Analytical sensitivity study showed that the assay could detect *M. tuberculosis* ranging from 3×10^5^ to 3.0 CFU per reaction with each of four probes. Among the 137 positive samples, one displayed an extremely low T_m_ in HEX-2. DNA sequencing result revealed a triple mutation (530 CTG>ATG/531 TCG>TTC), a variation not found previously among the local patients. These results demonstrate that TaqMan probe-based FMCA with color multiplexing can be directly used to scan the existence of unknown mutations within the amplicon.

### Multicolor FMCA for Mutation Identification

**Figure 4:**
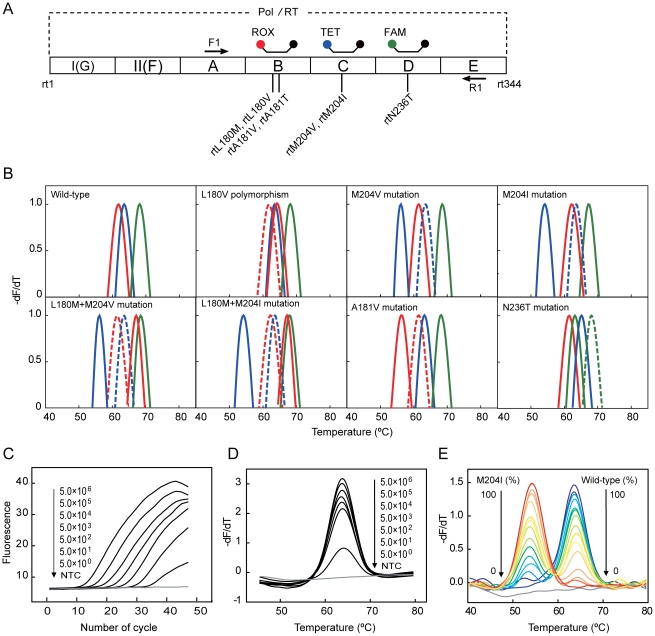
Mutation identification in HBV with 3-color PCR-MCA. **A**) The relative position of the primers and probes along the POL/RT region of HBV genome. Primers are shown as arrows and probes are shown by their respective labeling fluorophores (fluorophores are shown as colorful dots, quencher are shown as black dots). The mutation types are given with their relative positions shown as vertical lines. **B**) Representative melting curves of the mutation types detected among 165 serum samples. In order to visually compare the T*_m_* values of different mutation types, the melting curves showing the minus derivative of fluorescence intensity with respect to temperature were first normalized between 0 and 1, and then the data between 0.4 and 1 were normalized again and plotted at values between 0 and 1 according to ref 21. In each panel, the three solid lines (normalized melting curves) obtained from the corresponding three probes determine the exact mutation type or polymorphism, while the dotted line(s) indicate(s) the presumed melting curve of the corresponding wild-type. FAM-labeled probe: green line, TET-labeled probe: blue line, ROX-labeled probe: red line. **C**) Real-time PCR amplification curves of the 3-color PCR-MCA with wild-type HBV ranging from 5.0 to 5.0×10^6^ copies per reaction. **D**) Melting curves of the 3-color PCR-MCA corresponding to the amplification curves with wild-type HBV ranging from 5.0 to 5.0×10^6^ copies per reaction (from below to up). **E**) Melting curves of mimic HBV quasispecies samples with varied percentages (from 0, 3, 5, 10, 20, 30, 40, 50, 60, 70, 80, 90, 95, 97 to 100%) of the mutant type of M204I relative to the wild-type HBV templates. Both wild-type and M204I templates were from cloned plasmids and the total concentration was roughly 3×10^8^ copies per reaction. NTCs are shown by gray lines in C), D) and E).

In the second proof-of-principle study, we designed three differently labeled probes (Figure 4A) to identify six mutations of HBV associated with resistance to lamivudine and/or adefovir, the most commonly used nucleoside analogs for treatment of HBV infection. The melting curve profiles of 7 mutation- and polymorphism-containing plasmids were unequivocally differentiated (Figure 4B). Analytical sensitivity study showed that this assay could detect serially diluted plasmids ranging from 5.0 to 5.0×10^6^ copies per reaction with each of the three probes in both real-time PCR and FMCA detection formats (Figure 4C and 4D). When wild-type and mutant type were mixed, mutant DNA could be repeatedly detected in the percentage of 1∼10% (Figure 4E). The assay was used to analyze blind serum specimens from 165 patients including 99 treated patients and 66 untreated patients, among which 49.5% and 4.6% were detected to harbor lamivudine- and/or adefovir-resistant mutation(s), respectively, as confirmed by DNA sequencing of PCR amplicons (Table S5). The concordance rate between FMCA and sequencing results was 100%. These results demonstrate that the TaqMan probe-based FMCA with color multiplexing can be used to identify the exact mutation type with combined probes in one reaction.

### Multicolor FMCA for Mutation Genotyping

**Figure 5:**
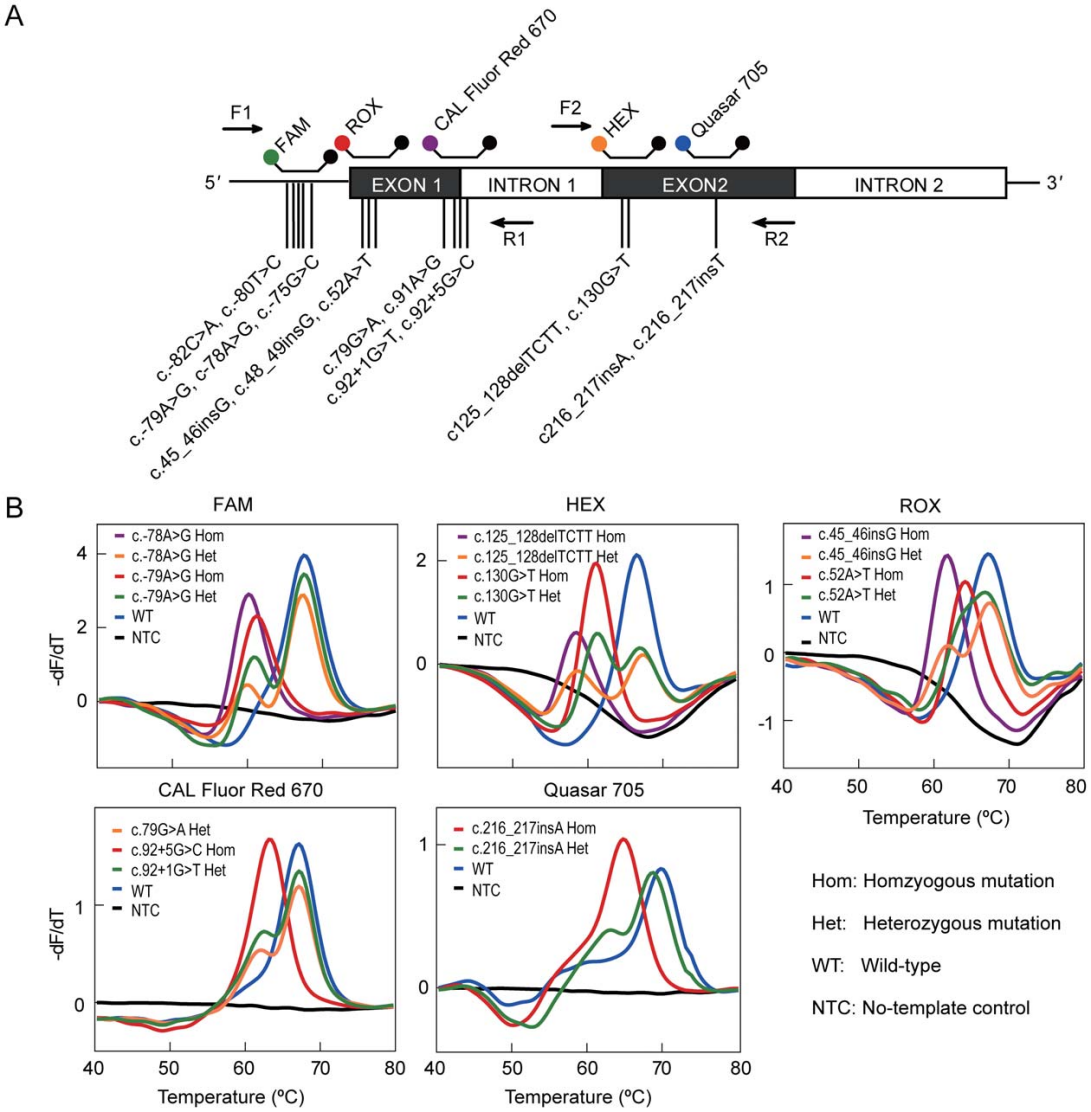
A 5-color FMCA assay for genotyping of 16 mutations in β-globin gene. **A**) The relative position of the primers and probes along the β-globin gene. Primers are shown as arrows and shared-stem molecular beacons are shown by their respective labeling fluorophores (fluorophores are shown as colorful dots, quencher are shown as black dots). The mutation types are given with their relative positions shown as vertical lines. **B**) Genotyping results of pre-characterized samples or plasmids. The derivative melting curves are displayed channel by channel indicated by the corresponding labeling fluorophores above the panel. For each sample, melting curves are shown in only one channel that reveals the occurrence of mutation together with the wild-type. The exact genotype is given by its mutation type.

In the third proof-of-principle study, we designed an FMCA assay to genotype 16 causative mutations of human β-thalassemia in *HBB* gene in a single tube. Based on the locations of the 16 mutations in *HBB*, five differently labeled probes were designed to be flanked by two pairs of primers (Figure 5A). The assay was first established with 18 pre-characterized samples and plasmids of known genotypes. The result showed that all heterozygous and homozygous mutant samples were correctly genotyped (Figure 5B). Analytical sensitivity study showed that the assay could correctly detect the genotypes from DNA concentrations ranging from 50 ng to 5.0 pg genomic DNA per reaction by all probes. A blind test of 94 clinical samples revealed 15 different genotypes, which were in 100% concordance with direct sequencing of PCR amplicons (Table S6). These data demonstrate that color multiplexing can be readily accomplished with shared-stem molecular beacons-based FMCA.

### Cross-platform Compatibility

To test the cross-platform compatibility of self-quenched probes-based FMCA, the *HBB* genotyping assay was performed on five types of commonly used real-time PCR instruments. The results showed that the 4-color genotyping assay could be accomplished on all of the five platforms. The five types of instruments actually differed in the program for melting curve analysis as well as in the format of data analysis. The time spent on melting analysis after PCR ranged from 15 min to 45 min. The absolute T_m_ values also varied in some genotype (within ±2°C). Nevertheless, the T_m_ value differences between the wild-type and mutant were similar across all platforms and the biggest difference was approximately 1°C (Table S7). In this testing, we also noticed that an off-line protocol, i.e., the PCR procedure ran independently on a regular thermocycler and FMCA was conducted separately on a real-time PCR machine, was feasible for FMCA. This off-line protocol permitted direct comparison of the melting analysis results from the same reaction on different platforms.

## Discussion

The distinct advantage of FMCA lies in its capability of multiplex mutation detection [27]. However, despite long and ongoing efforts towards enhanced multiplexing in FMCA, a combined merit of color multiplexing, flexible probe design, and cross-platform compatibility has never been achieved with a single probe type. In this regard, the dual-labeled, self-quenched probes described here represent further efforts towards that goal. Both TaqMan probes and shared-stem molecular beacons probes can be labeled with various fluorophores and can also be detected by most real-time PCR platforms. Therefore, color multiplexing and cross-compatibility are achieved with these probes. Because dual-labeled probes are already widely used in real-time PCR, their design and synthesis are well-established and the cost is lower than those with special modification. One peculiar requirement is asymmetric amplification, which is however easy to perform without extra cost. Thus, the dual-labeled, self-quenched probes offer a combined advantage of enhanced multiplexing, improved flexibility in probe design, and expanded cross-platform compatibility.

The reason that we chose these two dual labeled, self quenched probes is based on our understanding on the nature of FMCA. FMCA aims to detect the inherent T_m_ difference between the wild-type and the mutant, thus measurable T_m_ resolution rather than a yes-or-no result (as in real-time PCR detection) and appropriate rather than ultimate specificity are pursued for FMCA probe. According to our hypothesis, nearly all existing real-time PCR probes are qualified for FMCA. Real-time PCR probes can be categorized into three classes from structural perspective, i.e., single-stranded linear probe, hairpin probe, and double-stranded probes [28]. The single-stranded linear probe constitutes the largest portion, among which TaqMan probe is the mostly widely used and many derivative probes are basically modified from the TaqMan probe for enhanced specificity at the price of increased design complexity and extra synthesis cost. In the class of hairpin probe, the original molecular beacons are too specific for FMCA [29] so that the less specific shared-stem molecular beacons [24] were chosen. The double-stranded probe class is excluded simply because it exhibits no big fluorescence change upon thermal dissociation from their targets [30].

The combined advantage of the two self-quenched probes was demonstrated in the three proof-of-principles assays. In the first example of random mutation scanning, four probes could be aligned in a tiling format to cover the entire range of the targeted mutation hotspots. This represents a big improvement from a previous study that used dual hybridization probe in which only a limited number of mutations could be detected due to masking of the targeted region by the anchor probes [31]. Compared to the fluorescein-labeled, T_m_ multiplexing-based mutation scanning strategy, our assay eliminated the risk of T_m_ cross-talk between probes and could possibly cover more regions [8]. In the second example of mutation identification, by using three differently labeled TaqMan probes, 6 mutations could be accurately detected and differentiated. These mutations so far could only be differentiated by using reverse hybridization line probe assay [32]. In the third example of mutation genotyping, five differently labeled probes were able to genotype 16 mutations in a single reaction. This was a significant improvement from a previous report that analyzed 10 *HBB* mutations using three dual hybridization probe sets in three reactions [33]. Taken together, self-quenched probes demonstrate improved flexibility than existing probes in all the major application areas of FMCA. Moreover, the widened cross-platform compatibility was also achieved as demonstrated in the 4-color genotyping assay that could be run on five different real-time PCR platforms.

Since multiple probes are often required for FMCA to achieve multiplex detection, background fluorescence may exert impact on FMCA sensitivity. We observed that while TaqMan probe had higher hybridization efficiency, shared-stem molecular beacons had lower background when the probe type was exchanged in the proof-of-principle assays. Therefore, shared-stem molecular beacons may be preferred when multiple probes are used. MGB-modified linear probes, such as Pleiades probes have been reported to have low background. However, such modifications not only incurred difficulty in probe design as well as high cost in probe synthesis, but they also significantly increased the T_m_ of the probe [20].

As an end-point detection format of PCR by nature, FMCA requires excess single-stranded amplicon for probe hybridization to generate sufficient FMCA signal. That led to the use of asymmetric PCR amplification for FMCA. Asymmetric PCR amplification can also mitigate probe hydrolysis because less primer extension on the probe binding strand causes less probe hydrolysis. The effect of asymmetric amplification was reflected in the peak height of FMCA signal, which was proportional to the fluorescence intensity of the PCR product. By comparison, very weak FMCA signal could be detected from symmetric PCR product despite its strong end-point fluorescence. This end-point nature of FMCA detection allows combined use of conventional PCR machines for target amplification and real-time PCR machines for FMCA signal acquisition to achieve high sample throughput analysis. It may also guide future development of inexpensive device for FMCA.

Melting temperature is a physical parameter of nucleic acid hybrid. Under constant reaction conditions of heating rate, salt concentration and probe-target concentrations, T_m_ is highly reproducible. Recently, Chakravorty et al [34] demonstrated the feasibility of identifying a large number of different target sequences with a small number of probes by the combined use of T_m_ and color signature. However, multiplexing obtained by probe alone will be limited. To achieve higher order multiplexing, combination with multiplexed primer should be introduced in the future. Alternatively, when used with high-density PCR devices [35], multicolor FMCA may produce signature profiles that allow identification of millions of targets simultaneously. In any case, with the combined merit of color multiplexing, design flexibility, and cross-platform compatibility achieved by the probes described here, FMCA would find increasing applications in clinical diagnostics, translational research, and even discovery of new genomic variants.

## Materials and Methods

### Ethics Statement

All clinical samples collected had already been used for their original diagnosis purposes by the hospitals and were supplied for this study as on-shelf, coded specimens without any patient information or identifiers that could be used to decode patient information. Thus, the current study has been exempted from ethical approval by Ethics Committee on Human Studies in Xiamen University.

### Oligonucleotides, Clinical Samples and Plasmids

Primers and probes were designed with Primer Premier 5.0 (PREMIER Biosoft International, Palo Alto, CA), T_m_ Utility v1.3 (Idaho Technologies Inc., Salt Lake City, UT), and Oligo 6.0 (AVG Technologies Inc., Chelmsford, MA). All primers, probes, and target oligonucleotides were synthesized and PAGE-purified by Sangon (Shanghai, China) except for CAL Fluor Red 670- and Quasar 705-labeled probes, which were synthesized and HPLC-purified by Biosearch Technologies (Novato, CA). The purity of all probes was confirmed by mass spectrometry. The concentration of each oligonucleotide was determined by ND-1000 UV-Vis spectrophotometer (NanoDrop Technologies, Wilmington, DE). In FMCA, both the TaqMan and shared-stem molecular beacon were designed in the same way as in real-time PCR. Unwanted secondary structure in the probe should be avoided for better hybridization with its target. The mutation sites are basically located in the centre of the probe so as to obtain a Tm shift around 2–6°C as estimated by the software. All probes used for the three proof-of-principles assays were designed to match with wild-types except in the detection of lamivudine- and adefovir-resistant mutations in HBV, where probe for amino acid 180 matched with mutant type of M instead of the wild-type of L. The wild-type samples were always used as positive controls and water was used as no-template control (NTC) in these assays.

*M. tuberculosis* cultured strains after sterilization were provided by Xiamen Centre for Diseases Control and Prevention. Genomic DNA of each strain was extracted using AxyPrep™ Bacterial Genomic DNA Miniprep Kit (Axygen Biosciences, Union City, CA). DNA concentration of each sample was determined by the ND-1000 UV-VIS spectrophotometer unless otherwise noted. Serum samples of lamivudine- and adefovir-treated patients infected with hepatitis B virus (HBV) were collected from the Traditional Chinese Medicine Hospital of Xiamen. HBV DNA from serum samples was extracted and quantified using the HBV PCR-Fluorescence Test Kit (Zeesan Biotech, Xiamen, China). Coded whole blood samples of known genotypes were obtained from Xiamen Maternity and Child Health Care Hospital and Zhuhai Maternity and Child Health Care Hospital. Human genomic DNA was extracted from the whole blood sample by an automatic blood DNA extraction system (Lab-Aid 820, Zeesan Biotech, Xiamen, China).

Plasmids of β-thalassemia mutations of c.-79A>G, c.-78A>G, c.48_49insG, c.52A>T, c.92+5G>C, c.130G>T, c.125_128delTCTT, c.216_217insA, plasmids of HBV mutations of M204V, M204I, L180M, A181T, A181V, N236T, and HBV L180V polymorphism were all prepared by PCR-mediated *in vitro* mutagenesis [36] and their sequences were confirmed by bi-directional DNA sequencing.

### Denaturation of Hybrids Formed between Probes and Synthetic Oligonucleotides

Thermal denaturation of hybrids formed between probes and synthetic oligonucleotide targets was carried out in a Rotor-Gene™ 6000 real-time rotary analyzer (Corbett Research, Mortlake, Australia). Each 25-µL solution contained 0.2 µM probe, 0.4 µM target oligonucleotides in 1× SSP buffer [67 mM Tris-HCl, pH 8.8, 16 mM (NH_4_)_2_SO_4_, 0.01% (W/V) Tween-20] in the presence of 2.0 mM MgCl_2_. Thermal denaturation procedure started from 95°C for 1 min, 35°C for 2 min, and then followed by raising temperature from 35°C to 90°C at 1°C/step with 5 s stop between each step. Fluorescence was recorded at each step in the corresponding detection channel. The data obtained were plotted as fluorescence versus temperature as well as the negative derivative of fluorescence over temperature versus temperature. T_m_ values were identified by the peak position of the latter curve.

### Optimization of PCR Conditions for Probe-based FMCA

Experimental conditions investigated included symmetric PCR versus asymmetric PCR, three-temperature PCR versus two-temperature PCR, 5′-nuclease active Taq HS (*TaKaRa Taq*™ Hot Start Version, TaKaRa Inc., Dalian, China) versus 5′-nuclease-deficient Klentaq1 (Ab Peptides, In., St. Louis, MO). A three-temperature PCR was carried as follows: The 25-µL reactions contained 1×SSP buffer, 3.0 mM MgCl_2_, 200 µM dNTPs, 1.0 U Taq HS or Klentaq1, 0.4 µM each of forward and reverse primers (symmetric PCR) or 0.04 µM forward primer and 0.4 µM reverse primer (asymmetric PCR), 0.2 µM probe, and 5 µL of DNA template (5.0×10^5^ copies of plasmid DNA). Water was used as NTC. Real-time PCR and FMCA protocols started with a denaturation step of 3 min at 95°C, followed by 50 cycles of 95°C for 10 s, 58°C for 15 s, and 75°C for 20 s. Fluorescence was measured at 58°C. Melting curve analysis began with a denaturation step of 1 min at 95°C, a hybridization step of 5 min at 45°C, and followed by stepwise temperature increase from 45°C to 90°C at 1°C/step with 5 s stop between each step. A two-temperature format of the above experiments was performed by combining both annealing (58°C for 15 s) and extension (75°C for 20 s) steps into a single step of 60°C for 1 min. The amplified target was part of *recA* gene of *Vibrio cholerae*. The forward and reverse primers used were 5′-TGTGCGTTTATCGATGCCGAGCAC-3′ and 5′-GCTTTTGGTGTCAAAGCCGC-3′. The probe used was 5′-ROX-CCTGATACCGACGAGCAAGCACTGGA-BHQ1-3′.

### Scanning for Rifampin-resistant Mutations in *M. tuberculosis*

Two reaction were set up to scan the mutations randomly occurred within an 81-bp region of *rpoB* gene of *M. tuberculosis* that confer resistance to rifampin. Each 25-µL reaction contained 1×SSP buffer, 2.0 mM MgCl_2_, 80 µM dNTPs, 0.5 U Taq HS, a primer/probe mix containing one primer pair (0.1 µM/1.0 µM) and two differently labeled probes (0.1 µM each), and 5 µL of DNA template (0.5 ng *M. tuberculosis* genomic DNA, equivalent to 1.0×10^5^ copies). FMCA started with a denaturation step of 5 min at 95°C, 13 cycles of 95°C for 15 s, 70°C (with −1°C per cycle) for 20 s, and 1 min at 72°C, 42 cycles of 95°C for 15 s, 57°C for 20 s and 75°C for 25 s, followed by a denaturation step of 2 min at 95°C, a hybridization step of 2 min at 40°C, and a stepwise temperature increase from 45°C to 80°C at 1°C/step with 5 s stop between each step.

### Detection of lamivudine- and adefovir-resistant mutations in HBV

Each 25-µL reaction contained 1×SSP buffer, 2.5 mM MgCl_2_, 200 µM dNTPs, 1.0 U Taq HS, a primer/probe mix containing one primer pair (0.05 µM/1.0 µM) and 3 differently labeled probes (0.2 µM each), and 2.0 µL of HBV DNA or plasmid DNA (∼1.0×10^6^ copies). FMCA protocol started with a denaturation step of 3 min at 95°C, 45 cycles of 15 s at 95°C, 20 s at 52°C, and 1 min at 72°C, followed by a denaturation step of 1 min at 95°C, a hybridization step of 5 min at 35°C, and a stepwise temperature increase from 40°C to 85°C at 1°C/step with 5 s stop between each step. Real-time PCR signal was measured at 52°C.

### Genotyping of β-Thalassemia Mutations

Each 25-µL reaction contained 1×SSP buffer, 2.5 mM MgCl_2_, 200 µM dNTPs, 1.0 U Taq HS, a primer/probe mix containing two primer pairs (0.1 µM/1.0 µM each) and 5 differently labeled probes (0.2 µM each), and 5 µL of human genomic DNA (50 ng) or plasmid DNA (∼5.0×10^6^ copies). FMCA protocols started with a denaturation step of 3 min at 95°C, 50 cycles of 15 s at 95°C, 10 s at 55°C, and 20 s at 76°C, followed by a denaturation step of 1 min at 95°C, hybridization step of 5 min at 35°C, and a stepwise temperature increase from 40°C to 80°C at 1°C/step with 5 s stop between each step.

### Cross-platform Compatibility Study

The above β-thalassemia mutation genotyping assay was used to evaluate the cross-platform compatibility of FMCA. Since Quasar 705 channel is not available in part of the platforms to be evaluated, the Quasar 705-labeled probe was omitted from the assay. Identical PCR condition was used in all instruments but the melting curve program was adapted to each machine. Five real-time PCR instruments, Rotor-Gene™ 6000 real-time rotary analyzer (Corbett Research, Mortlake, Australia), CFX 96™ real-time PCR detection system (Bio-Rad, Hercules, CA), ABI 7500 (Life Technologies, Carlsbad, CA), Stratagene Mx3005P (Agilent, Santa Clara, CA), and LightCycler® 480 (Roche Applied Sciences, Indianapolis, IN) were tested. Fifteen DNA samples representing 15 genotypes were used for this study. Wild-type sample was used as positive control and water was used as NTC.

## Supporting Information

Figure S1 **Derivative melting curves of four TaqMan probes of 26 nt, 30 nt, 36 nt and 41 nt with their respective oligonucleotide targets.** For each probe, melting curves from high to low T*m* correspond to targets from 1 to n, where “n” stands for the number of the targets for each probe (Table S3). The targets have differently mismatched nucleotides with their probe. The black lines represent the melting curves of the probes in the absence of the targets. Differently colored lines represent targets with different mismatches to the hybridization probe, with fully matched, wild-type target giving the highest T*m* value (positioned at the far right side of the peak).
(TIF)

Figure S2 **Comparison of symmetric and asymmetric PCR in FMCA.** **A**) Symmetric PCR. **B**) Asymmetric PCR. A two-temperature cycling protocol was performed using either 5′-nuclease active Taq HS (blue lines) or 5′-nuclease-deficient Klentaq1 (red lines) DNA polymerase. Three data forms are given from left to right: amplification curves, meting curves, and negative derivative melting curves. Probe cleavage is shown by the fluorescence difference between the blue and black lines (indicated by a bracket). No-template controls are shown in black lines.
(TIF)

Table S1 Melting curves of TaqMan probe with different targets.
(DOC)

Table S2 Melting curves of shared-stem molecular beacon probe with different targets.
(DOC)

Table S3 TaqMan probes of 26 nt, 30 nt, 36 nt and 41 nt and their corresponding targets.
(DOC)

Table S4 The 2-color mutation scanning assay results of 311 samples.
(DOC)

Table S5 HBV mutation types identified from 164 clinical samples.
(DOC)

Table S6 The 5-color genotyping assay results of 94 samples.
(DOC)

Table S7 Cross-platform comparison of FMCA results.
(DOC)
