# Supplementary figures and images for: Multiplex Fluorescence Melting Curve Analysis for Mutation Detection with Dual-Labeled, Self-Quenched Probes
Source: PLoS One. 2011 Apr 28;6(4):e19206. doi: 10.1371/journal.pone.0019206 (PMC3084284; doi:10.1371/journal.pone.0019206)

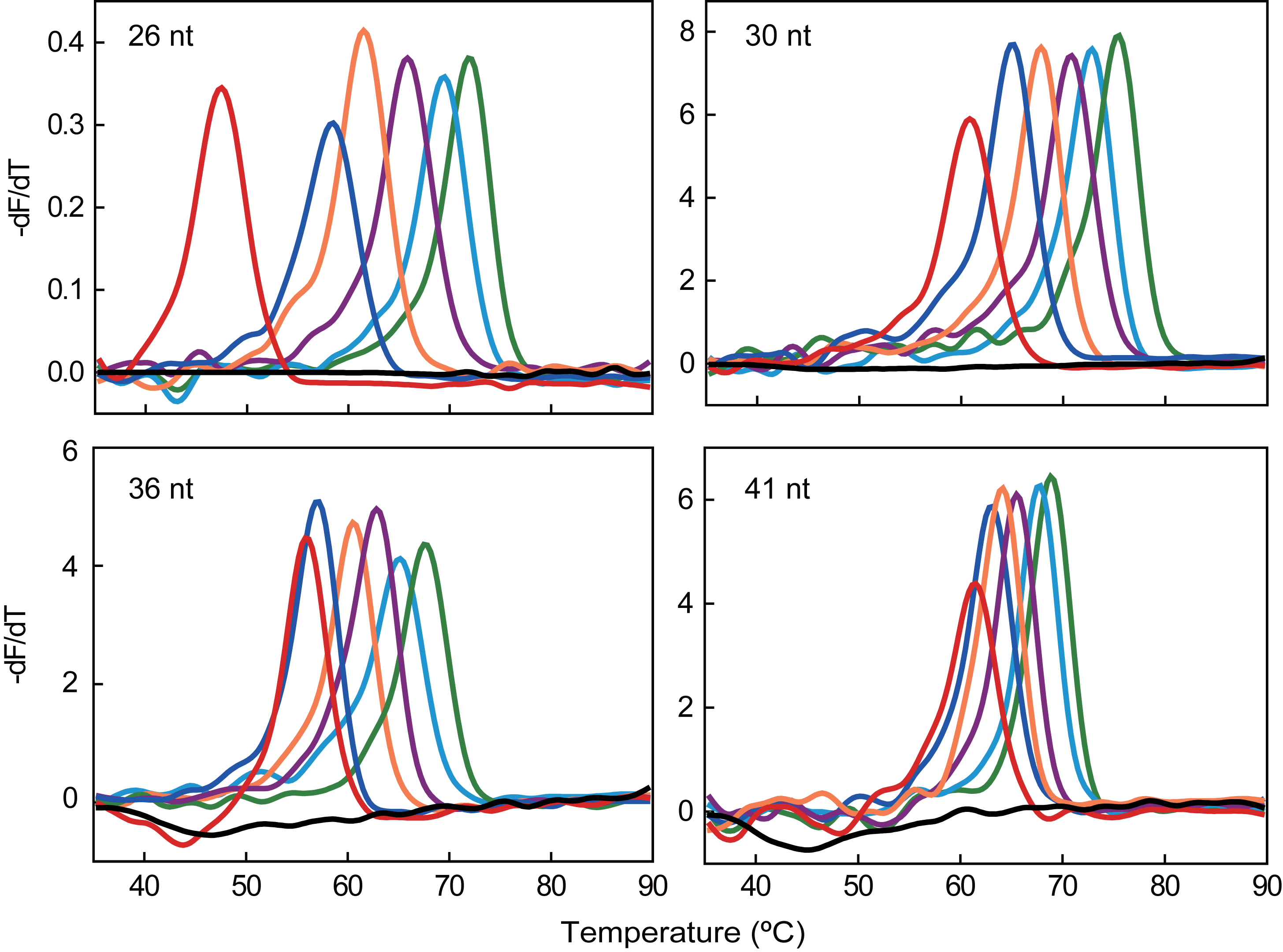

Supplement: Figure S1 — Derivative melting curves of four TaqMan probes of 26 nt, 30 nt, 36 nt and 41 nt with their respective oligonucleotide targets. For each probe, melting curves from high to low Tm correspond to targets from 1 to n, where “n” stands for the number of the targets for each probe (Table S3). The targets have differently mismatched nucleotides with their probe. The black lines represent the melting curves of the probes in the absence of the targets. Differently colored lines represent targets with different mismatches to the hybridization probe, with fully matched, wild-type target giving the highest Tm value (positioned at the far right side of the peak). (TIF) [file pone.0019206.s001.tif]

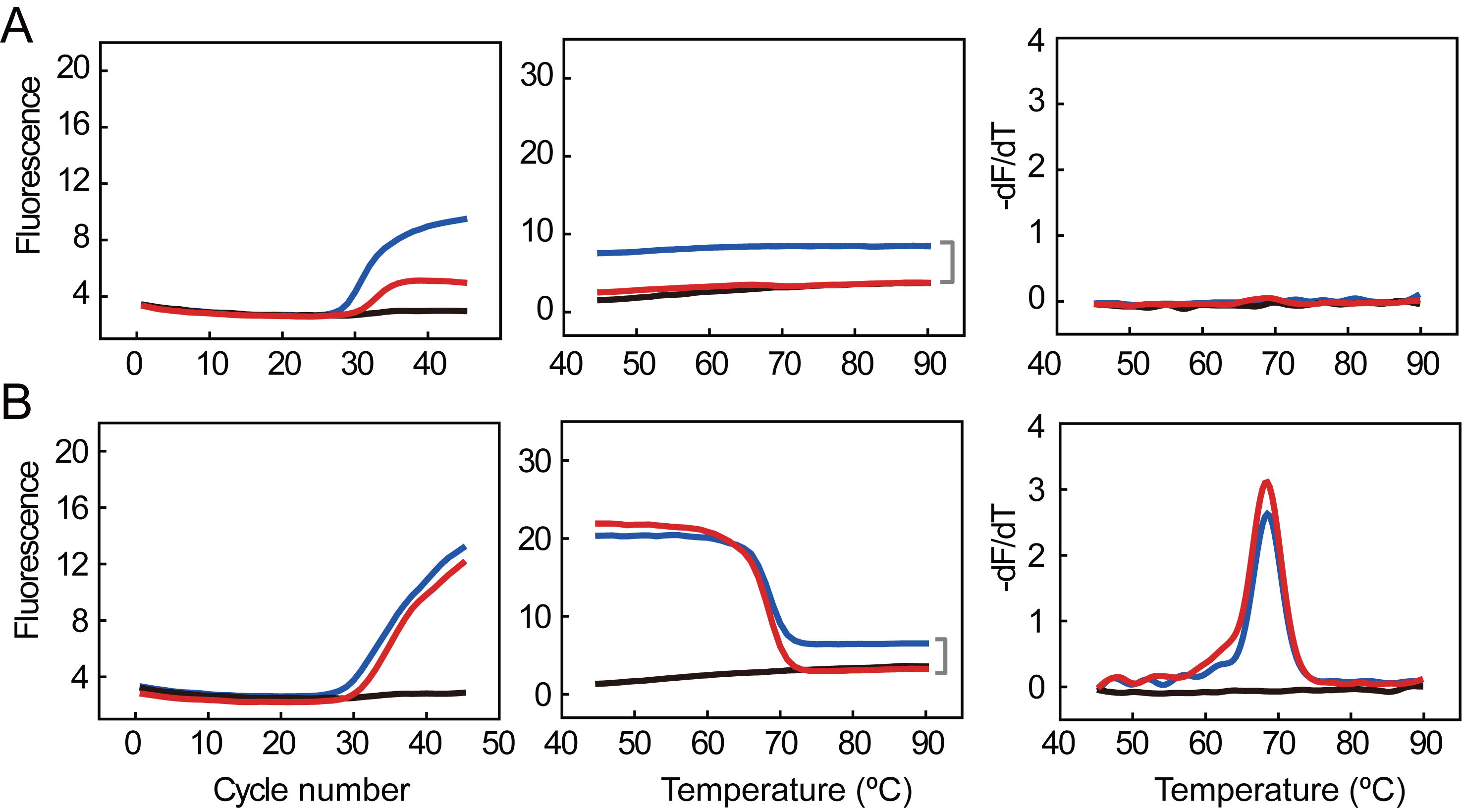

Supplement: Figure S2 — Comparison of symmetric and asymmetric PCR in FMCA. A) Symmetric PCR. B) Asymmetric PCR. A two-temperature cycling protocol was performed using either 5′-nuclease active Taq HS (blue lines) or 5′-nuclease-deficient Klentaq1 (red lines) DNA polymerase. Three data forms are given from left to right: amplification curves, meting curves, and negative derivative melting curves. Probe cleavage is shown by the fluorescence difference between the blue and black lines (indicated by a bracket). No-template controls are shown in black lines. (TIF) [file pone.0019206.s002.tif]
