# Supplementary material for: Multiplex Fluorescence Melting Curve Analysis for Mutation Detection with Dual-Labeled, Self-Quenched Probes
Source: PLoS One. 2011 Apr 28;6(4):e19206. doi: 10.1371/journal.pone.0019206 (PMC3084284; doi:10.1371/journal.pone.0019206)
Supplement: Table S1 — Melting curves of TaqMan probe with different targets. (DOC) [file pone.0019206.s003.doc]

| **Table S1.** Melting curves of TaqMan probe with different targets | | |
| --- | --- | --- |
| Oligonucleotides | Sequences (5'→3') ^a^ | T*_m_*(°C) |
| TaqMan probe | TET-TGGTGCGGTAGATGTGATTGTTGTTG-BHQ |  |
| Target 1 | CAACAACAATCACATCTACCGCACCA | 72.5 |
| Target 2 | CAACGACAATCACATCTACCGCACCA | 71.0 |
| Target 3 | CAACAACAATCACATCTACTGCACCA | 68.5 |
| Target 4 | CAACAACGATAACATCTACCGCACCA | 65.5 |
| Target 5 | CAACGACGATGACATCTACCGCACCA | 62.0 |
| Target 6 | CAACAACGATGACATCCACAGCACCA | 52.0 |
| ^a^The underlined nucleotides represent the variant sites. | | |
