# Supplementary material for: Multiplex Fluorescence Melting Curve Analysis for Mutation Detection with Dual-Labeled, Self-Quenched Probes
Source: PLoS One. 2011 Apr 28;6(4):e19206. doi: 10.1371/journal.pone.0019206 (PMC3084284; doi:10.1371/journal.pone.0019206)
Supplement: Table S2 — Melting curves of shared-stem molecular beacon probe with different targets. (DOC) [file pone.0019206.s004.doc]

| **Table S2.** Melting curves of shared-stem molecular beacon probe with different targets | | |
| --- | --- | --- |
| Oligonucleotides | Sequences (5'→3')^a^ | T*_m_*(°C) |
| Probe | FAM-CGGCTGGGCATAAAAGTCAGGGCCG-BHQ |  |
| Target 1 | GCTGCCCTGACTTTTATGCCCAGCCCTG | 70.0 |
| Target 2 | GCTGCCCTGACTTTTAGGCCCAGCCCTG | 66.5 |
| Target 3 | GCTGCCCTGAGTTTTATGCCCAGCCCTG | 65.5 |
| Target 4 | GCTGCCCTGACTTTCATGCCCAGCCCTG | 63.5 |
| Target 5 | GCTGCCCTGACTTCTATGCCCAGCCCTG | 62.5 |
| Target 6 | GCTGCCCTGACTTTTATTCCCAGCCCTG | 60.0 |
| ^a^The underlined nucleotides represent the variant sites. | | |
