# Supplementary material for: Multiplex Fluorescence Melting Curve Analysis for Mutation Detection with Dual-Labeled, Self-Quenched Probes
Source: PLoS One. 2011 Apr 28;6(4):e19206. doi: 10.1371/journal.pone.0019206 (PMC3084284; doi:10.1371/journal.pone.0019206)
Supplement: Table S3 — TaqMan probes of 26 nt, 30 nt, 36 nt and 41 nt and their corresponding targets. (DOC) [file pone.0019206.s005.doc]

| **Table S3.** TaqMan probes of 26 nt, 30 nt, 36 nt and 41 nt and their corresponding targets | |
| --- | --- |
| Probes/targets | Sequences (5'→3')^a^ |
| 26-nt probe  target 1  target 2  target 3  target 4  target 5  target 6 | ROX-CCTGATACCGACGAGCAAGCACTGGA-BHQ1  ATTTCCAGTGCTTGCTCGCCGGTATCAGGCTG  ATTTCCAGTGCTTGCTCGCCGGTATCTGGCTG  ATTTCCAGTGCTTGCTCGCCAGTATCAGGCTG  ATTTCCAGCGCTTGTTCGCCGGTATCAGGCTG  ATTTCTAGCGCTTGCTCGCCGGTATCTGGCTG  ATTTCCAGCGCTTGTTCACCTGTATCAGGTTG |
| 30-nt probe  target 1  target 2  target 3  target 4  target 5  target 6 | ROX-CACTGGAAATTTGTGATGCATTGGCTCGCT-BHQ1  AACGACAATCACATCTACCGCACCAGAGCGAGCCAATGCATCACAAATTTCCAGTGC  AACAACAATCACATCTACCGCACCAGAGCGAGCCAGTGCATCACAAATTTCCAGTGC  AACAACAATCACATCTACCGCACCAGAGCGAGCCAATGCATCACAAATCTCCAGTGC  AACAACAATCACATCTACTGCACCAGAGCGAGCTAGTGCATCACAAATTTCCAGCGC  AACAACAATCACATCTACCGCACCAGAGCGAGCCAGCGCATCACAGATTTCCAGCGC  AACGACGATGACATCTACCGCACCAGAGCGAGCCAGCGCATCACAGATTTCTAGCGC |
| 36-nt probe  target 1  target 2  target 3  target 4  target 5  target 6 | TET-ATTAAGCAGATGCGTTTTCCCGGTTACTTCTTGATC-BHQ1  AACTCCATCACGATCAAGAAGTAACCGGGAAAACCCATCTGGTTTATCACATCGAGCTC  AACTCCATCACGATCAAGAAGTAACCGGGAAAGCCCATCTGGTTAATCACATCGAGCTC  AACTCCATCACGATCAAGAAGTAGCCGGGAAAGCCCATCTGGTTAATCACATCGAGCTC  AACTCCATCACGATCAAGAAGTATCCGGGAAAGCCCATCTGGTTAATCACATCGAGCTC  AACTCCATCACGACCAAGAAGTATCCGGGAAAGCCCATCTGGTTAATCACATCGAGCTC  AATTCCATCACGATCAAGAAATAGCCGGGAAAGCCCATCTGGTTGATGACATCGAGCTC |
| 41-nt probe  target 1  target 2  target 3  target 4  target 5  target 6 | ROX- TGTGATTAACCAGGCTTTCCCGATTACTTCTTGATCGTGAT-BHQ1  AACTCCATCACGATCAAGAAGTAACCGGGAAAGCCCATCTGGTTAATCACATCGAGCTC  AACTCCATCACGATCAAGAAGTATCCGGGAAAGCCCATCTGGTTAATCACATCGAGCTC  AACTCCATTACGATCAAGAAGTATCCGGGAAAGCCCATCTGGTTAATCACATCGAGCTC  AACTCCATCACGACCAAGAAGTATCCGGGAAAGCCCATCTGGTTAATCACATCGAGCTC  AACTCCATCACGATCAAGAAGTAACCGGGAAAACCCATCTGGTTTATCACATCGAGCTC  AATTCCATCACGATCAAGAAATAGCCGGGAAAGCCCATCTGGTTGATGACATCGAGCTC |
| ^a^The nucleotides in the shadow represent the probe binding sequences and the underlined nucleotides indicate the variant sites. | |
