# Supplementary material for: Multiplex Fluorescence Melting Curve Analysis for Mutation Detection with Dual-Labeled, Self-Quenched Probes
Source: PLoS One. 2011 Apr 28;6(4):e19206. doi: 10.1371/journal.pone.0019206 (PMC3084284; doi:10.1371/journal.pone.0019206)
Supplement: Table S4 — The 2-color mutation scanning assay results of 311 samples. (DOC) [file pone.0019206.s006.doc]

| **Table S4.** The 4-color mutation scanning assay results of 311 samples | | | | | | |
| --- | --- | --- | --- | --- | --- | --- |
| Type | ΔT*_m_* (°C)^a^ | | | | Sequencing result | No. |
|  | Quasar 670 | HEX | FAM | ROX |  |  |
| 1 | / | / | / | / | Wild-type | 174 |
| 2 | / | 6.1 | / | / | 516 GAC>TAC | 1 |
| 3 | / | 4.2 | / | / | 516 GAC>GTC | 2 |
| 4 | / | 1 | / | / | 516 GAC>GGC | 1 |
| 5 | / | / | 4.2 | / | 522 TCG>TTG | 2 |
| 6 | / | / | 4.3 | / | 526 CAC>AAC | 2 |
| 7 | / | / | 3.1 | / | 526 CAC>CGC | 5 |
| 8 | / | / | 4.2 | / | 526 CAC>CTC | 2 |
| 9 | / | / | 5.1 | / | 526 CAC>GAC | 6 |
| 10 | / | / | 4.2 | / | 526 CAC>GGC | 1 |
| 11 | / | / | 5.1 | / | 526 CAC>TAC | 3 |
| 12 | / | / | 6.1 | / | 526 CAC>TGC | 3 |
| 13 | / | / | / | 4.2 | 531 TCG>TTG | 93 |
| 14 | / | / | / | 7.4 | 531 TCG>TGG | 1 |
| 15 | / | / | / | 0.0^c^ | 533 CTG>CCG | 3 |
| 16 | 5.1 | 7.2 | / | / | 511 CTG>CCG / 515 ATG>ATC | 1 |
| 17 | 5.1 | 1.0 | / | / | 511 CTG>CCG / 516 GAC>GGC | 4 |
| 18 | 6.0 | / | 5.0 | / | 511 CTG>CCG / 526 CAC>CAA | 1 |
| 19 | / | 3.0 | 4.1 | / | 515 ATG>AGG / 526 CAC>AAC | 1 |
| 20 | / | / | 4.2 | 0.0^c^ | 526 CAC>CAG / 533 CTG>CCG | 1 |
| 21 | 0.0^b^ | / | 5.0 | / | 512 AGC>WGC / 526 CAC>GAC | 1 |
| 22 | / | / | 5.2 | 0.0^b^ | 526 CAC>SAC / 531 TCG>TYG | 1 |
| 23 | / | / | 5.0 | / | 526 CAC>YAC / 526 CAC>CGC | 1 |
| 24 | / | / | / | 19.5 | 530 CTG>ATG / 531 TCG>TTC | 1 |

^a^ΔT*_m_* = T_1_- T_2_, where T_1_ represents T*_m_* of the wild-type and T_2_ indicates T*_m_* of the mutant.

^b^The assumed mutant peak is merged with the wild-type peak, indicating the mutant is minor whereas the wild-type is abundant.

^c^A unique melting curve without a peak was given by 533 CTG > CCG.
