# Supplementary material for: Multiplex Fluorescence Melting Curve Analysis for Mutation Detection with Dual-Labeled, Self-Quenched Probes
Source: PLoS One. 2011 Apr 28;6(4):e19206. doi: 10.1371/journal.pone.0019206 (PMC3084284; doi:10.1371/journal.pone.0019206)
Supplement: Table S5 — HBV mutation types identified from 164 clinical samples. (DOC) [file pone.0019206.s007.doc]

**Table S5.** HBV mutation types identified from 164 clinical samples

| Sample type^a^ | Treated patients^b^ | **Untreated patients^c^** |
| --- | --- | --- |
| Wild-type (including L180V) | 50 | **62** |
| Lamivudine-resistant | 37 | **1** |
| M204I | 18 | **0** |
| M204V+L180M | 9 | **0** |
| M204I+L180M | 2 | **0** |
| M204I+Wild-type | 3 | **1** |
| M204V+L180M+Wild-type | 4 | **0** |
| M204I+L180M+Wild-type | 1 | **0** |
| Adefovir-resistant | 11 | **2** |
| A181V | 2 | **1** |
| N236T | 1 | **1** |
| A181V+N236T | 1 | **0** |
| N236T+Wild-type | 4 | **0** |
| A181V+N236T+Wild-type | 1 | **0** |
| M204I+N236T | 2 | **0** |
| lamivudine- and adefovir-resistant | 1 | **1** |
| L180M+M204V+N236T | 1 | **0** |
| **Mutation rate (%)** | **49.5** | **4.6** |

^a^Mutation types include quasispecies as shown by more than one types. ^b^Treated patients represent those who have been treated with nucleoside analogs. ^c^Untreated patients are nucleoside analogs naïve.
