# Supplementary material for: Multiplex Fluorescence Melting Curve Analysis for Mutation Detection with Dual-Labeled, Self-Quenched Probes
Source: PLoS One. 2011 Apr 28;6(4):e19206. doi: 10.1371/journal.pone.0019206 (PMC3084284; doi:10.1371/journal.pone.0019206)
Supplement: Table S6 — The 5-color genotyping assay results of 94 samples. (DOC) [file pone.0019206.s008.doc]

| **Table S6.** The 5-color genotyping assay results of 94 samples | | | | | | |
| --- | --- | --- | --- | --- | --- | --- |
| Genotype | Number | Channel | T_1_ (°C)/T_2_ (°C)^a^ | | | ΔT_m_(°C)^b^ |
| Wild-type and heterozygous carriers | | | | | | |
| Wild-type | 10 | / | | / | / | |
| c.-78A>G | 14 | FAM | | 67.5 / 61.0 | 6.5 | |
| c.-79A>G | 2 | FAM | | 67.3 / 60.0 | 7.3 | |
| c.45_46insG | 2 | ROX | | 67.5 / 64.2 | 3.3 | |
| c.52A>T | 14 | ROX | | 67.5 / 62.0 | 5.5 | |
| c.79G>A | 3 | CY5 | | 67.2 / 62.0 | 5.2 | |
| c.92+1G>T | 2 | CY5 | | 67.2 / 62.5 | 4.7 | |
| c.125_128delTCTT | 23 | HEX | | 67.3 / 58.7 | 8.6 | |
| c.130G>T | 4 | HEX | | 67.0 / 61.3 | 5.7 | |
| c.216_217insA | 11 | Quasar 705 | | 67.0 / 62.6 | 4.4 | |
| Homozygous and compound heterozygous mutations | | | | | | |
| c.[-78A>G ]+[-78A>G] | 2 | FAM | | 67.5 / 61.0 | 6.5 | |
| c.[125_128delTCTT]+[125_128delTCTT] | 2 | HEX | | 67.3 / 58.7 | 8.6 | |
| c.[125_128delTCTT]+[-78A>G] | 2 | HEX | | 67.3 / 58.7 | 8.6 | |
|  |  | FAM | | 67.5 / 61.0 | 6.5 | |
| c.[125_128delTCTT]+[79G>A] | 1 | HEX | | 67.3 / 58.7 | 8.6 | |
|  |  | CY5 | | 67.2 / 62.0 | 5.2 | |
| c.[125_128delTCTT]+[216_217insA] | 2 | HEX | | 67.3 / 58.7 | 8.6 | |
|  |  | Quasar 705 | | 67.0 / 62.6 | 4.4 | |
| ^a^ T_1_: T*_m_* of the wild-type, T_2_: T*_m_* of the mutant; ^b^ ΔT*_m_* = T_1_- T_2_. | | | | | | |
