# Supplementary material for: Multiplex Fluorescence Melting Curve Analysis for Mutation Detection with Dual-Labeled, Self-Quenched Probes
Source: PLoS One. 2011 Apr 28;6(4):e19206. doi: 10.1371/journal.pone.0019206 (PMC3084284; doi:10.1371/journal.pone.0019206)
Supplement: Table S7 — Cross-platform comparison of FMCA results. (DOC) [file pone.0019206.s009.doc]

| **Table S7.** Cross-platform comparison of FMCA results. | | | | | | | | | | | |
| --- | --- | --- | --- | --- | --- | --- | --- | --- | --- | --- | --- |
| **Genotype** | **Channel** | **Rotorgen-6000** | | **LightCycler 480** | | **CFX96** | | **ABI 7500** | | **Mx3000P** | |
|  |  | **T_1_/T_2_ (°C)^a^** | **ΔT_m_(°C)^b^** | **T_1_/T_2_ (°C)^a^** | **ΔT_m_(°C)^b^** | **T_1_/T_2_ (°C)^a^** | **ΔT_m_(°C)^b^** | **T_1_/T_2_ (°C)^a^** | **ΔT_m_(°C)^b^** | **T_1_/T_2_ (°C)^a^** | **ΔT_m_(°C)^b^** |
| Heterozygous mutation | | | | | | | | | | | |
| c.-78A>G | FAM | 67.5 / 61.0 | 6.50 | 66.62 / 60.26 | 6.36 | 66.50 / 60.00 | 6.50 | 66.50 / 59.70 | 6.80 | 67.00 / 60.20 | 6.80 |
| c.-79A>G | FAM | 67.3 / 60.0 | 7.30 | 67.52 / 59.53 | 7.99 | 66.50 / 59.00 | 7.50 | 66.50 / 58.90 | 7.60 | 67.00 / 59.51 | 7.49 |
| c.45_46insG | ROX | 67.5 / 64.2 | 3.30 | 67.72 / 64.57 | 3.15 | 66.00 / 63.00 | 3.00 | 66.20 / 63.00 | 3.20 | 67.67 / 64.18 | 3.49 |
| c.52A>T | ROX | 67.5 / 62.0 | 5.50 | 67.55 / 61.97 | 5.58 | 66.00 / 60.50 | 5.50 | 66.20 / 60.80 | 5.40 | 67.65 / 62.42 | 5.23 |
| c.79G>A | CY5 | 67.2 / 62.0 | 5.20 | 68.09 / 63.23 | 4.86 | 65.50 / 61.00 | 4.50 | 66.90 / 61.80 | 5.10 | 67.76 / 62.95 | 4.81 |
| c.92+1G>T | CY5 | 67.2 / 62.5 | 4.70 | 67.92 / 63.35 | 4.57 | 66.00 / 62.00 | 4.00 | 66.30 / 62.00 | 4.30 | 67.63 / 63.58 | 4.05 |
| c.125_128delTCTT | HEX | 67.3 / 58.7 | 8.60 | 66.42 / 58.71 | 7.71 | 65.00 / 57.50 | 7.50 | 65.70 / 57.10 | 8.60 | 66.90 / 59.20 | 7.70 |
| c.130G>T | HEX | 67.0 / 61.3 | 5.70 | 66.70 / 61.24 | 5.46 | 65.00 / 59.50 | 5.50 | 65.10 / 59.30 | 5.80 | 66.50 / 61.14 | 5.36 |
| Homozygous and compound heterozygous mutation | | | | | | | | | | | |
| c.[-78A>G ]+[-78A>G] | FAM | 67.5 / 61.0 | 6.50 | 66.96 / 60.54 | 6.42 | 66.50 / 60.00 | 6.50 | 66.50 / 59.70 | 6.80 | 66.96 / 60.23 | 6.73 |
| c.[-79A>G]+[-79A>G] | FAM | 67.3 / 60.0 | 7.30 | 66.96 / 59.17 | 7.79 | 66. 50 / 59.00 | 7.50 | 66.50 / 58.90 | 7.60 | 66.96 / 59.51 | 7.45 |
| c.[45_46insG]+[45_46insG] | ROX | 67.5 / 64.2 | 3.30 | 67.83 / 64.57 | 3.26 | 66. 50 / 63.50 | 3.00 | 66.10 / 63.00 | 3.10 | 67.68 / 64.72 | 2.96 |
| c.[52A>T]+[52A>T] | ROX | 67.5 / 62.0 | 5.50 | 67.83 / 62.26 | 5.57 | 66.00 / 60.50 | 5.50 | 66.10 / 60.80 | 5.30 | 67.80 / 62.08 | 5.72 |
| c.[92+5G>T]+[92+5G>T] | CY5 | 67.2 / 63.5 | 3.70 | 67.92 / 64.04 | 3.88 | 66.00 / 62.00 | 4.00 | 66.30 / 62.20 | 4.10 | 67.76 / 63.88 | 3.88 |
| c.[125_128delTCTT]+[125_128delTCTT] | HEX | 67.3 / 58.7 | 8.60 | 66.42 / 58.82 | 7.60 | 65.00 / 57.50 | 7.50 | 65.70 / 57.10 | 8.60 | 66.90 / 59.24 | 7.66 |
| c.[130G>T]+[130G>T] | HEX | 67.0 / 61.3 | 5.70 | 66.42 / 61.13 | 5.29 | 65.00 / 59.50 | 5.50 | 65.40 / 59.70 | 5.70 | 66.50 / 61.14 | 5.36 |
| ^a^ T_1_: T*_m_* of the wild-type, T_2_: T*_m_* of the mutant; ^b^ ΔT*_m_* = T_1_ - T_2_. | | | | | | | | | | | |
